# Textbook outcomes in liver surgery for gallbladder cancer patients treated with curative-intent resection: a multicenter observational study

**DOI:** 10.1097/JS9.0000000000000510

**Published:** 2023-06-05

**Authors:** Zhi-Peng Liu, Wei Guo, Da-Long Yin, Wei-Yue Chen, Jiao-Yang Wang, Xue-Lei Li, Ping Yue, Chao Yu, Zhao-Ping Wu, Rui Ding, Yi Zhu, Fan Huang, Jin-Xue Zhou, Dong Zhang, Wei Chen, Yan Jiang, Jie Bai, Jing-Jing Wang, Yan-Qi Zhang, Hai-Su Dai, Wan Yee Lau, Zhi-Yu Chen

**Affiliations:** a Department of Hepatobiliary Surgery, Southwest Hospital, Third Military Medical University (Army Medical University), Chongqing, China; b Department of Hepatobiliary Surgery, Capital Medical University Affiliated Beijing Friendship Hospital, Beijing, China; c Department of Hepatobiliary Surgery, The First Affiliated Hospital of USTC, Hefei, China; d Clinical Research Center of Oncology, Lishui Hospital of Zhejiang University, Lishui, China; e Department of Hepatobiliary Surgery, Lanzhou University First Affiliated Hospital, Lanzhou, China; f Department of Hepatobiliary Surgery, The Affiliated Hospital of Guizhou Medical University, Guizhou, China; g Department of Hepatobiliary Surgery, Jiujiang First People's Hospital, Jiujiang, China; h Department of Hepatobiliary Surgery, Xijing Hospital, Fourth Military Medical University (Air Force Medical University), Xi'an, China; i Department of Hepatobiliary Surgery, Zhejiang University School of Medicine Second Affiliated Hospital, Hangzhou, China; j Department of Hepatobiliary Surgery, First Affiliated Hospital of Anhui Medical University, Hefei, China; k Department of Hepatobiliary Pancreatic Surgery, Henan Provincial Tumor Hospital, Zhengzhou, China; l Department of Hepatobiliary Surgery, Xi 'an Jiaotong University Medical College First Affiliated Hospital, Xi 'an, China; m Department of Hepatobiliary Surgery, Sun Yat-sen University First Affiliated Hospital, Zhongshan, China; n Department of Health Statistics, College of Military Preventive Medicine, Third Military Medical University (Army Medical University), Chongqing, China; o Faculty of Medicine, the Chinese University of Hong Kong, Shatin, New Territories, Hong Kong SAR, China

**Keywords:** gallbladder neoplasms, resection, short-term prognosis, prediction, textbook outcomes in liver surgery

## Abstract

**Background:**

Cholecystectomy, hepatectomy, and lymphadenectomy are recommended as the curative treatment for resectable gallbladder cancer (GBC). Textbook outcomes in liver surgery (TOLS) is a novel composite measure that has been defined by expert consensus to represent the optimal postoperative course after hepatectomy. This study aimed to determine the incidence of TOLS and the independent predictors associated with TOLS after curative-intent resection in GBC patients.

**Methods:**

All consecutive GBC patients who underwent curative-intent resection between 2014 and 2020 were enrolled from a multicenter database from 11 hospitals as the training and the internal testing cohorts, and Southwest Hospital as the external testing cohort. TOLS was defined as no intraoperative grade greater than or equal to 2 incidents, no grade B/C postoperative bile leaks, no postoperative grade B/C liver failure, no 90-day postoperative major morbidity, no 90-day readmission, no 90-day mortality after hospital discharge, and R0 resection. Independent predictors of TOLS were identified using logistic regression and were used to construct the nomogram. The predictive performance was assessed using the area under the curve and calibration curves.

**Results:**

TOLS was achieved in 168 patients (54.4%) and 74 patients (57.8%) from the training and internal testing cohorts, and the external testing cohort, respectively. On multivariate analyses, age less than or equal to 70 years, absence of preoperative jaundice (total bilirubin≤3 mg/dl), T1 stage, N0 stage, wedge hepatectomy, and no neoadjuvant therapy were independently associated with TOLS. The nomogram that incorporated these predictors demonstrated excellent calibration and good performance in both the training and external testing cohorts (area under the curve: 0.741 and 0.726).

**Conclusions:**

TOLS was only achieved in approximately half of GBC patients treated with curative-intent resection, and the constructed nomogram predicted TOLS accurately.

## Graphical abstract

**Figure:**
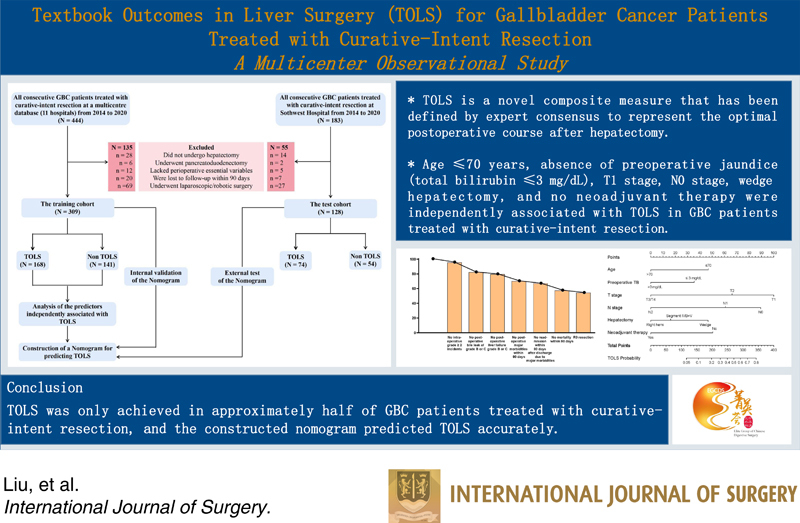

## Introduction

Highlights

- Textbook Outcomes in Liver Surgery (TOLS) is a novel composite measure that has been defined by expert consensus to represent the optimal postoperative course after hepatectomy.
- TOLS was only achieved in approximately half of gallbladder cancer (GBC) patients treated with curative-intent resection, which consisted of cholecystectomy, liver resection, and lymphadenectomy.
- Age less than or equal to 70 years, absence of preoperative jaundice (total bilirubin≤3 mg/dl), T1 stage, N0 stage, wedge hepatectomy, and no neoadjuvant therapy were independently associated with TOLS in GBC patients treated with curative-intent resection.
- A nomogram was constructed in predicting TOLS for GBC patients treated with curative-intent resection.

Gallbladder cancer (GBC), accounting for ~80% of all biliary tract tumors, is common^1^. In 2018, there were more than 200 000 new cases of GBC worldwide^2^. Curative resection is the only treatment that can cure this disease. In 2021, the National Comprehensive Cancer Network (NCCN) updated the surgical treatment to include cholecystectomy, hepatectomy, and lymphadenectomy for all patients with resectable GBC^3^. This operation can provide long-term survival outcomes, but the complexity of surgery can result in perioperative morbidity and mortality rates which range from 16.2 to 25.0%, and from 0.7 to 1.6%, respectively^4^. Previous reported studies on short-term outcomes for GBC patients after surgery focuced on single outcomes of morbidity and mortality, which do not reflect the actual assssment of the quality of the surgical procedure from the patient’s perspective^5,6^.

For any patient undergoing a complex surgery, a comprehensive and accurate assessment of the short-term outcomes is very important, and is conducive to the improvement of surgical strategies and decision-making^7^. Although a single outcome can still be used to improve in certain specific ways, there are many disadvantages of using a single outcome in an individual patient^5,6^. In addition, the use of an individual outcome to compare hospital performance across institutions is not comprehensive. Therefore, using a composite outcome to assess the short-term outcomes of surgery is better^8,9^. Textbook outcome (TO) is a novel, patient-centered composite outcome that aggregates all the desirable short-term outcomes^10^. If a patient achieves all these desirable outcomes, then the patient has achieved the most desirable or TO. In addition, TO not only can be used to assess the short-term prognosis of an individual, but it can also be used to compare the performance of different institutions^11^. However, TO in the past was usually defined by a single expert or a small group of surgeons, making it less authoritative and limited its general acceptance. Görgec *et al*. conducted an international single-round survey among all members of the European-African and International Hepato-Pancreato-Biliary Associations to propose the first international definition of TO in Liver Surgery (TOLS)^12^, which provides a standardized criteria to evaluate patients undergoing liver surgery worldwide.

With the change in the recommended method of curative resection for GBC, the number of patients who undergo hepatectomy in addition to cholecystectomy and lymphadenectomy for curative resection will increase significantly. However, TOLS has not been applied to assess the short-term outcomes for GBC patients. To our knowledge, this is the first study to use TOLS to assess the surgical quality in GBC patients treated with curative-intent resection. Specifically, this study determined the incidence of TOLS and the independent predictors associated with TOLS by using a multicenter database maintained prospectively by 11 professional hepatobiliary centers on GBC patients treated with curative-intent resection. In addition, this study constructed and tested a nomogram in predicting the probability of achieving TOLS in GBC patients treated with curative-intent resection.

## Methods

### Patient selection

The data of this study came from a prospectively maintained multicenter database and from Southwest Hospital, which were used as the training and internal testing cohorts, and the external testing cohort, respectively. The database of the training and internal testing cohorts came from 11 tertiary hospitals in China, including Capital Medical University Affiliated Beijing Friendship Hospital, The First Affiliated Hospital of USTC, Lanzhou University First Affiliated Hospital, The Affiliated Hospital of Guizhou Medical University, Jiujiang First People’s Hospital, Xijing Hospital, Zhejiang University School of Medicine Second Affiliated Hospital, First Affiliated Hospital of Anhui Medical University, Henan Provincial Tumor Hospital, Xi’an Jiaotong University Medical College First Affiliated Hospital, Sun Yat-sen University First Affiliated Hospital. The external testing cohort came from Southwest Hospital.

The same inclusion and exclusion criteria were used in all these cohorts. All the consecutive patients were treated with curative-intent resection for a newly diagnosed GBC from January 2014 to January 2020. The diagnosis of GBC was confirmed by postoperative pathological examinations. The cohorts excluded patients who a) did not undergo hepatectomy; b) underwent pancreaticoduodenectomy; c) lacked perioperative essential variables; d) were lost to follow-up within 90 days after hospital discharge; and e) underwent laparoscopic/robotic surgery. Approval for this study was obtained from the Ethics Committees of all the hospitals. All patients provided written informed consent, Supplemental Digital Content 2, http://links.lww.com/JS9/A591 prior to receiving treatment. This retrospective study was registered with ResearchRegistry.com (Unique Identification Number: researchregistry8541). Data has been reported in line with STROCSS 2021 criteria^13^, Supplemental Digital Content 1, http://links.lww.com/JS9/A590.

### Surgical procedure

Based on the NCCN recommendations, curative-intent resection included at least cholecystectomy, hepatectomy, and lymph node dissection^3^. There were three types of hepatectomy, namely right hemihepatectomy, segment 4B+5 resection, and wedge hepatectomy. Right hemihepatectomy was defined as resection of Couinaud’s liver segments 5–8, based on the H5678 definition of the ‘New world’ hepatectomy terminology^14^. Segment 4B+5 resection was defined as anatomic resection of Couinaud’s liver segments 4B+5^15^. Wedge hepatectomy was defined as hepatectomy of the gallbladder fossa with a 3~4 cm margin in the liver^15^. Additional procedures, such as vascular reconstruction, common bile duct resection, hepaticopancreaticoduodenectomy, and choledochojejunostomy were based on the discretion of the surgical team according to the extent of tumor invasion. Each operating surgeon had the experience of more than 100 hepatectomies per year.

### Data collection

The data in the multicenter databases was collected prospectively, and maintained dynamically. The data was analyzed retrospectively for the training and internal testing cohorts to include age, sex, American Society of Anesthesiologists score, comorbidity, preoperative jaundice, tumor discovery time, total bilirubin (TB), albumin, alanine aminotransferase (ALT), international normalized ratio (INR), carcinoembryonic antigen, carbohydrate antigen 19-9, adenocarcinoma tumor size, peripheral nerve invasion, tumor differentiation, intraoperative blood loss, type of hepatectomy, bile duct procedure, adjuvant therapy, neoadjuvant therapy, and 8th American Joint Committee on Cancer (AJCC) staging^16^. The upper or lower limit of normal values was used to divide patients into the normal and high/low groups, including 35 g/l for albumin, 40 U/l for ALT, 1.15 for INR, 5 ug/ml for carcinoembryonic antigen, and 37 U/l for carbohydrate antigen 19-9. Preoperative jaundice was defined as preoperative TB>54 µmol/l (3 mg/dl)^17^.

### Definition of TOLS

The definition of TOLS was based on the International Expert Delphi Consensus in Liver Surgery, to include: no intraoperative grade greater than or equal to 2 incidents (as defined by the Oslo classification)^18^, no postoperative grade B/C bile leak (as defined by the International Study Group of Liver Surgery)^19^, no postoperativegrade B/C liver failure (as defined by the International Study Group of Liver Surgery)^20^, no postoperative major morbidity (Clavien–Dindo grade III–IV) within 90 days^21^, no readmission within 90 days due to surgery-related major morbidity (Clavien–Dindo grade ≥ III), no mortality within 90 days after hospital discharge, and R0 resection^12^. R0 resection was defined as a histological tumor-free margin ≥ 1 mm. Only when all the seven predetermined outcomes were achieved was the patient considered to have achieved TOLS. The sources of all clinical history could be divided into two parts: during hospitalization, all clinical history was sourced from the medical comments and nursing comments; from discharge to 90 days after discharge, all clinical history was sourced from outpatient records, consultation, and phone call.

### Statistical analysis

Continuous values were expressed as mean±SD or median (quartile), The student *t* test or Mann–Whitney *U* test was used as appropriate. Categorical variables were expressed as numbers and percentages, and the χ^2^ test or Fisher’s exact test was used as appropriate. In the training and testing cohorts, all patients were divided into the TOLS and non TOLS groups, and baseline data between groups were compared. A line graph was used to reflect the distribution and probability of achieving TOLS in the training cohort. The probability of achieving TO is calculated separately for patients treated with wedge hepatectomy, segment IVB + V resection, and right hemi hepatectomy in the training cohort. Univariate and multivariate analyses were performed using logistic regression with forward stepwise variable selection to identify independent predictors associated with TOLS. In the multivariate logistic regression analyses, only variables with a *P* value<0.10 in the univariate analyses were included. To choose the nomogram factors, variables independently associated with TOLS and were clinically accessible to construct the nomogram were selected. The predictive performance in the training and testing cohorts was assessed using discrimination and calibration. The area under the receiver operating characteristic (ROC) curve was used to calculate the area under curve (AUC) to measure the discrimination. The calibration plot was assessed using the Hosmer–Lemeshow test. The predictive performance of the nomogram was compared with the 8th AJCC stage using ROC curves and decision curve analysis (DCA) in the training and testing cohorts. DCA displayed the true- and false-positive fractions as functions of the risk threshold, which compensated for the deficiency of the ROC curves. SPSS 26.0 (SPSS) and R software (version 3.5.2. http://www.r-project.org/) were used for statistical analysis in this study. An online nomogram was constructed for better clinical application and promotion. A *P* value<0.05 with a two-tailed test was considered to indicate a significant difference.

## Results

### Patients selection

**Figure 1:**
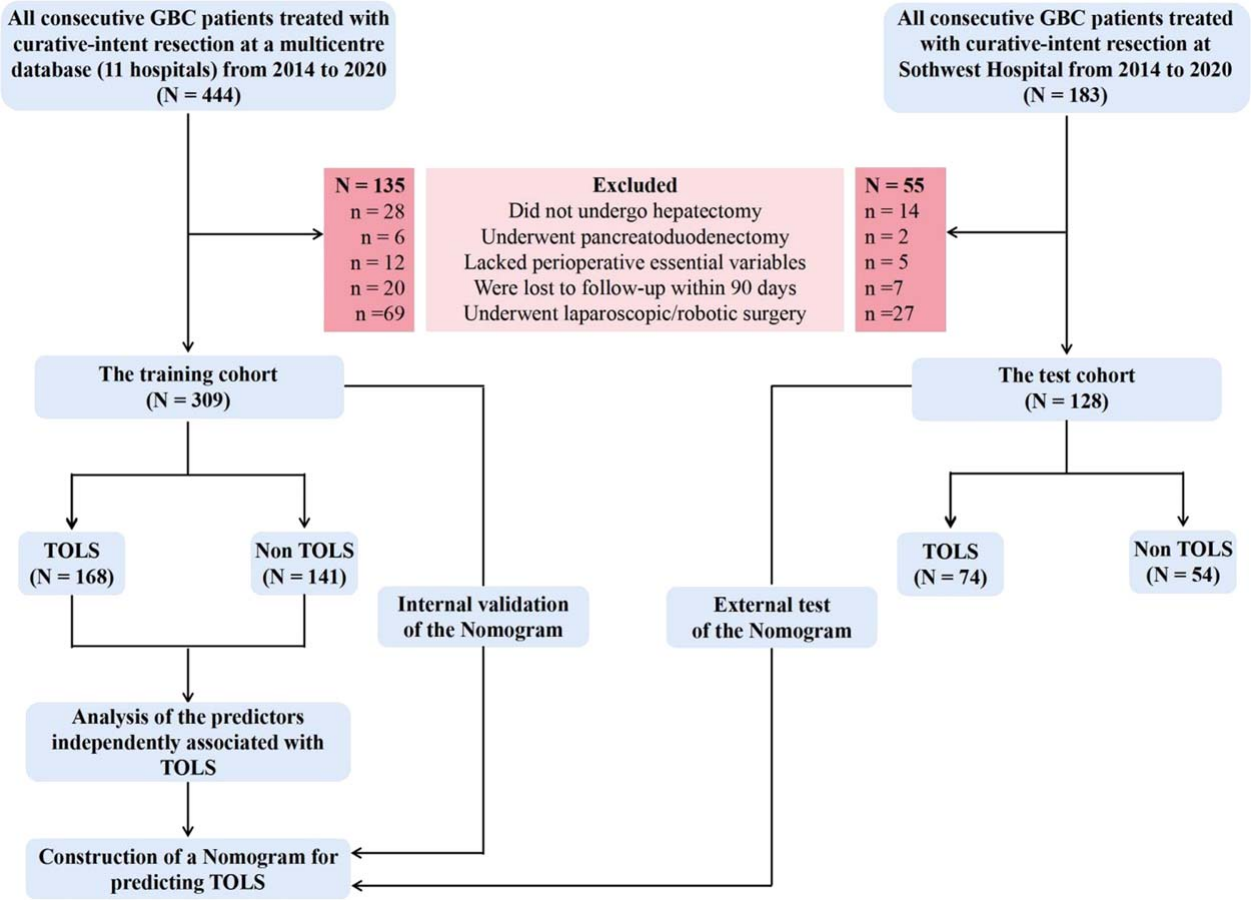
Patient selection for GBC. GBC, gallbladder cancer; TOLS, Textbook outcomes in Liver Surgery.

The study process and patient selection are shown in Figure 1. There were 309 patients in the training and internal testing cohorts and 128 patients in the external testing cohort. In the training cohort, 120 patients were males (38.8%) with the mean±SD age of 63.0±10.5 years, 76 patients had preoperative jaundice (24.6%), the median tumor size was 26.0 (15.0, 40.0) mm, 33 patients had T3/T4 stage GBC (10.7%), 43 patients had N2 stage GBC (13.9%), 213 patients underwent wedge hepatectomy (68.9%), 57 patients underwent segment 4B+5 resection (18.4%), and 139 patients underwent right hemihepatectomy (12.6%).

### Distribution of TOLS

**Figure 2:**
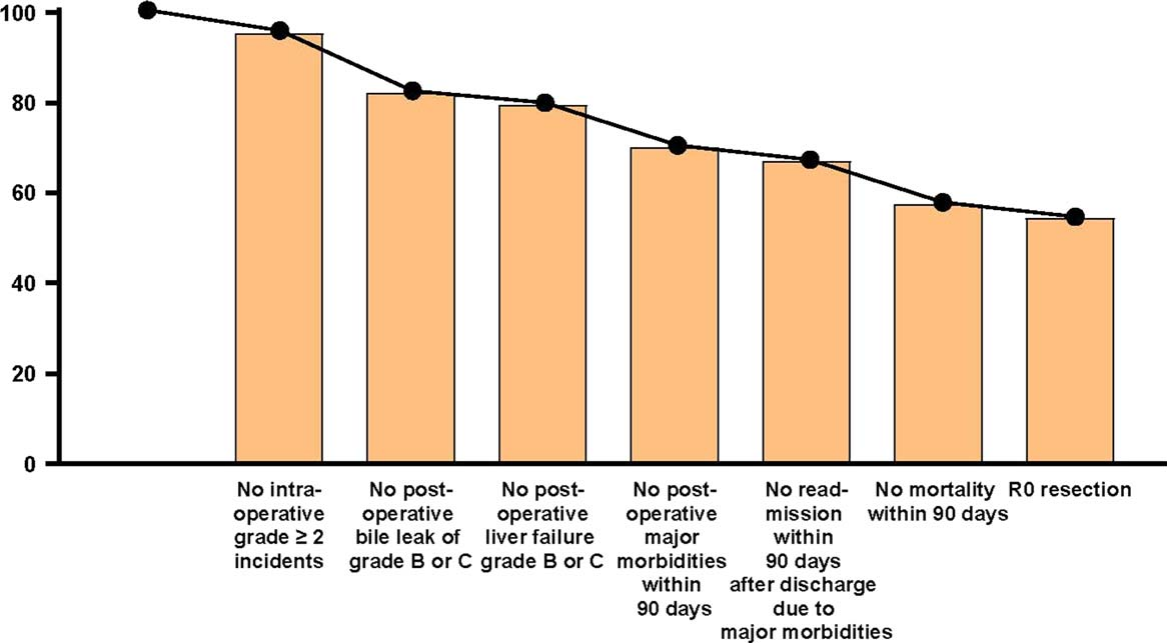
Distribution of TOLS for GBC from the training cohort.

In both the training and external testing cohorts, the percentage achieved for each short-term outcome was calculated, as shown in Supplemental Table 1, Supplemental Digital Content 3, http://links.lww.com/JS9/A593. TOLS was achieved in 168 patients (54.4%) in the training cohort and in 74 patients (57.8%) in the external testing cohort. There were no postoperative major morbidities within 90 days in 74.4% of patients, no postoperative grade B or C liver failure in 96.8%, no readmission within 90 days after hospital discharge due to major morbidities in 94.5%, and no intraoperative grade greater than or equal to 2 incidents in 95.5% (Fig. 2). In addition, the probabilities of achieving TO were 59.2 (126/213), 49.1 (28/57), and 35.9 (14/39), respectively, for patients treated with wedge hepatectomy, segment IVB + V resection, and right hemi hepatectomy in the training cohort.

### Predictors associated with TOLS

The baseline characteristics between the TOLS and non TOLS groups in the training cohort are shown in Table 1. Fewer TOLS patients had preoperative jaundice, lower levels of TB, ALT, and INR, higher levels of albumin, earlier T, N, and AJCC tumor stages, a larger extent of hepatectomy, and fewer patients with neoadjuvant therapy. The baseline characteristics between the TOLS and non TOLS groups in the external testing cohort are shown in Supplemental Table 2, Supplemental Digital Content 4, http://links.lww.com/JS9/A594. The results of univariate and multivariate logistic regression analyses for TOLS in the training cohort are shown in Table 2. On multivariate logistic regression analyses, age less than or equal to 70 years, absence of preoperative jaundice (TB≤3 mg/dl), T1 stage disease, N0 stage disease, wedge hepatectomy, and no neoadjuvant therapy were independently associated with TOLS in GBC patients treated with curative-intent resection.

**Table 1:** Baseline characteristics for GBC between TOLS and Non TOLS group in training cohort.

Variables	Total (*N*=309)	TOLS (*N*=168)	Non TOLS (*N*=141)	*P*
Age, years^a^	61.4±10.5	61.1±10.4	61.8±10.7	0.580
Male	120 (38.8)	71 (42.3)	49 (34.8)	0.177
ASA score>II grade	20 (6.5)	7 (4.2)	13 (9.2)	0.072
Comorbidity	99 (32.0)	52 (31.0)	47 (33.3)	0.655
Preoperative jaundice	76 (24.6)	28 (16.7)	48 (34.0)	<0.001
Preoperative PTCD	23 (7.4)	14 (8.3)	9 (6.4)	0.515
Incidentally discovered	31 (10.0)	13 (7.7)	18 (12.8)	0.143
TB, umol/ml^a^	15.3 (10.8, 41.3)	14.7 (10.9, 22.5)	17.6 (10.7, 76.7)	0.049
Albumin, g/l^a^	39.6±5.8	40.3±6.0	38.7±5.4	0.012
ALT, U/l^a^	35.0 (17.8, 88.5)	28.0 (17.1, 64.3)	41.3 (18.0, 113.5)	0.012
INR^a^	0.99±0.17	0.98±0.90	1.01±0.23	0.071
CEA, ug/ml^a^	2.8 (1.7, 5.4)	2.7 (1.6, 4.6)	2.9 (1.9, 6.0)	0.130
CA 19-9, U/l^a^	29.9 (9.3, 194.6)	22.2 (9.7, 131.6)	43.2 (8.3, 300.2)	0.109
Tumor size, mm^a^	26.0 (15.0, 40.0)	24.5 (16.0, 40.0)	27.0 (15.0, 40.0)	0.482
Poor differentiation	104 (33.7)	53 (31.5)	51 (36.2)	0.392
Adenocarcinoma	258 (83.5)	142 (84.5)	116 (82.3)	0.595
8th AJCC T stage				<0.001
T1	93 (30.1)	57 (33.9)	36 (25.5)	
T2	183 (59.2)	104 (61.9)	79 (56.0)	
T3/T4	33 (10.7)	7 (4.2)	26 (18.4)	
8th AJCC N stage				<0.001
N0	174 (56.3)	113 (67.3)	61 (43.3)	
N1	92 (29.8)	41 (24.4)	51 (36.2)	
N2	43 (13.9)	14 (8.3)	29 (20.6)	
8th AJCC staging system				<0.001
I stage	56 (18.1)	41 (24.4)	15 (10.6)	
II stage	103 (33.3)	60 (35.7)	43 (30.5)	
III stage	106 (34.3)	52 (31.0)	54 (38.3)	
IV stage	44 (14.2)	15 (8.9)	29 (20.6)	
Type of hepatectomy				0.019
Wedge hepatectomy	213 (68.9)	126 (75.0)	87 (61.7)	
Segment IVB + V resection	57 (18.4)	28 (16.7)	29 (20.6)	
Right hemi hepatectomy	39 (12.6)	14 (8.3)	25 (17.7)	
Intraoperative blood loss, ml^a^	300 (200, 500)	300 (200, 400)	300 (200, 600)	0.451
Bile duct procedure^b^	142 (46.0)	73 (43.5)	69 (48.9)	0.335
Adjuvant therapy	92 (29.8)	52 (31.0)	40 (28.4)	0.621
Neoadjuvant therapy	47 (15.2)	15 (8.9)	32 (22.7)	0.001

a Note: Continuous values are expressed as the mean±SD or median (quartile).

b The bile duct procedure included common bile duct resection and cholangiojejunostomy.

AJCC, American Joint Committee on Cancer; ALT, alanine aminotransferase; ASA, American Society of Anesthesiologists; CA 19-9, carbohydrate antigen 19-9; CEA, carcinoembryonic antigen; INR, international normalized ratio; TB, total bilirubin.

**Table 2:** Univariate and multivariate logistic regression analyses for TOLS in the training cohort.

		Univariate analyses		Multivariable analyses^a^	
Variables	Comparison	OR (95% CI)	*P*	OR (95% CI)	*P*
Age	≤70 *vs.*>70 (years)	2.215 (1.218–3.831)	0.004	2.365 (1.275–4.387)	0.006
Sex	Female *vs.* Male	0.728 (0.458–1.155)	0.178		
ASA score	≤II *vs.*>II (grade)	2.336 (0.905–6.026)	0.079	2.457 (0.855–7.061)	0.095
Comorbidity	No *vs.* Yes	1.115 (0.691–1.801)	0.655		
Preoperative jaundice	No *vs.* Yes	2.321 (1.362–3.955)	0.002	2.004 (1.085–3.701)	0.026
Preoperative PTCD	Yes *vs.* No	1.333 (0.559–3.180)	0.516		
Incidentally discovered	No *vs.* Yes	1.745 (0.823–3.700)	0.147		
Albumin	≥ 35 *vs.*<35 (g/L)	2.302 (1.240–4.276)	0.008	1.497 (0.710–3.155)	0.289
ALT	≤40 *vs.*>40 (U/L)	1.779 (1.128–2.807)	0.013	1.093 (0.601–1.990)	0.770
INR	≤1.15 *vs.*>1.15	2.489 (0.830–7.460)	0.104		
CEA	≤5 *vs.*>5 (ug/ml)	1.552 (0.934–2.578)	0.090	0.961 (0.509–1.812)	0.901
CA 19-9	≤37 *vs.*>37 (U/L)	1.701 (1.081–2.677)	0.022	0.994 (0.536–1.665)	0.843
Tumor size	≤30 *vs.*>30 (mm)	1.049 (0.664–1.657)	0.838		
Tumor differentiation	Well/Moderate *vs.* Poor	1.230 (0.766–1.974)	0.392		
Adenocarcinoma	Yes *vs.* No	1.177 (0.645–2.148)	0.595		
8th AJCC T stage	T2a/T2b *vs.* T3/T4	1.203 (0.723–2.002)	0.478	1.767 (0.864–3.613)	0.119
	T1 *vs.* T3/T4	5.881 (2.313–14.951)	<0.001	6.747 (2.245–20.276)	0.001
8th AJCC N stage	N1 *vs.* N2	1.529 (0.918–2.547)	0.103	1.734 (0.961–3.129)	0.068
	N0 *vs.* N2	3.308 (1.631–6.710)	0.001	5.245 (2.128–12.925)	<0.001
Type of hepatectomy	Segment IVB+V resection *vs.* Right hemi hepatectomy	1.500 (0.834–2.697)	0.176	1.788 (0.935–3.418)	0.079
	Wedge hepatectomy *vs.* Right hemi hepatectomy	2.586 (1.273–5.255)	0.009	2.559 (1.119–5.848)	0.026
Intraoperative blood loss	≤300 *vs.*>300 (ml)	1.413 (0.893–2.234)	0.140		
Bile duct procedure^b^	No *vs.* Yes	1.247 (0.796–1.955)	0.336		
Adjuvant therapy	No *vs.* Yes	0.883 (0.541–1.444)	0.621		
Neoadjuvant therapy	No *vs.* Yes	2.994 (1.547–5.796)	0.001	2.745 (1.325–5.689)	0.007

a Note: The variables found to be significant at *P*<0.10 in univariate analyses were entered into multivariate Cox regression analyses.

b The bile duct procedure included common bile duct resection and cholangiojejunostomy.

AJCC, American Joint Committee on Cancer; ALT, alanine aminotransferase; ASA, American Society of Anesthesiologists; CA 19-9, carbohydrate antigen 19-9; CEA, carcinoembryonic antigen; INR, international normalized ratio; OR, odds ratio; TB, total bilirubin.

### Development and validation of the nomogram for TOLS

**Figure 3:**
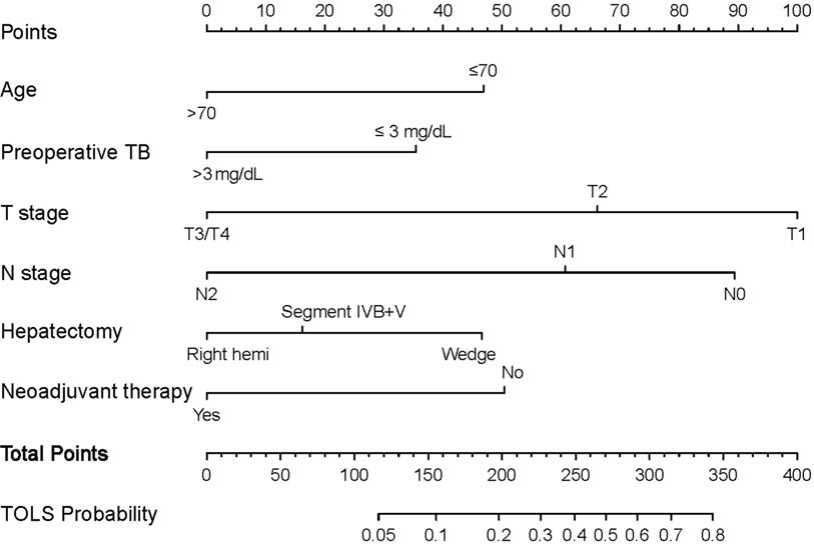
Nomogram for predicting TOLS in GBC treated with curative-intent resection patients. TB, total bilirubin; TOLS, Textbook outcome in liver surgery.

**Figure 4:**
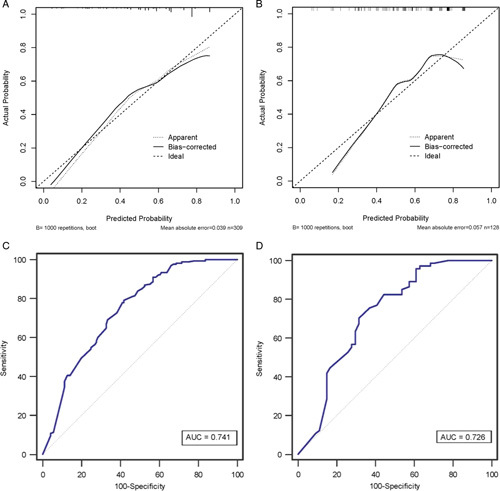
Applicability verified using calibration and ROC curve of the model in the training (A and C) and test (B and D) cohorts. AJCC, American Joint Committee on Cancer; AUC, area under the curve.

Using the independent variables derived from the multivariate analysis, a nomogram to predict the probability that a patient would experience TOLS after curative-intent resection of GBC was constructed (Fig. 3). Each variable in the nomogram was assigned a score, and the total score of the nomogram was obtained by adding the scores obtained for each of these variables. A vertical line was then drawn downward to obtain a total score, which represented the probability of a patient in achieving TOLS. The score for each factor and the calculation formula are shown in Supplemental Table 3, Supplemental Digital Content 5, http://links.lww.com/JS9/A604. For better promotion in clinical practice, the nomogram is available online at the following free website: https://liupredictionmodel.shinyapps.io/GBC_TOLS/. In both the training and external testing cohorts, the nomogram demonstrated good calibration for risk estimation, (Figs. 4A and 4B) and good accuracy in assessing the probability of TOLS, with an AUC of 0.741 (95% CI: 0.689–0.789) in the training cohort and 0.726 (95% CI: 0.640–0.801) in the external testing cohort (Figs. 4C and 4D). The optimal nomogram cutoff value was 288, with the corresponding probability of TOLS being 58.4%. The sensitivity and specificity for the nomogram in predicting TOLS were 79.2 and 58.2%, respectively, in the training cohort, and 70.3 and 68.5%, respectively, in the external testing cohort.

### Comparison of predictive ability of different models

**Figure 5:**
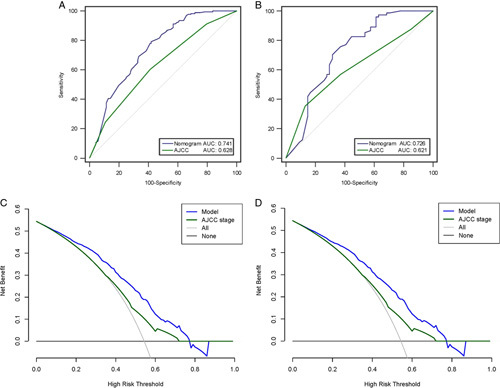
Model applicability compared with the 8th AJCC stage using receiver operating characteristic ROC curve and decision curve analysis in the training (A and C) and test (B and D) cohorts. AJCC, American Joint Committee on Cancer; AUC, area under the curve.

The AUC of the nomogram was significantly higher than the AUC of the eighth AJCC staging in both the training (0.741 versus 0.628, *P*<0.001) and the external testing (0.726 versus 0.621, *P*<0.001) cohorts (Figs. 5A and 5B). The DCA curve revealed that the range of the probability thresholds of the nomogram was higher than that of the eighth AJCC stage. Thus, the nomogram was more beneficial than the eighth AJCC stage in predicting the probability of TOLS for GBC (Figs. 5C and 5D). The detailed comparison of the predictive abilities for TOLS between the nomogram and the eighth AJCC stage is shown in Supplement Table 4, Supplemental Digital Content 6, http://links.lww.com/JS9/A606.

## Discussion

The NCCN recommends since 2021 that all resectable GBC patients be treated with cholecystectomy, hepatectomy, and lymphadenectomy for curative resection^3^. As this recommendation increases the number of patients to undergo hepatectomy for GBC, surgeons need a better measure to assess the short-term outcomes of this operation. TOLS, a new composite measure has been defined by the International Expert Delphi Consensus to represent the most ideal short-term outcome for patients undergoing hepatectomy^12^. To our knowledge, this is the first study to use TOLS as a composite measure to assess the short-term outcomes in GBC patients after curative-intent resection. Our study showed the contribution of each of the outcome measures in achieving TOLS can vary greatly: while most patients could achieve no intraoperative grade greater than or equal to 2 incidents, no postoperative liver failure grade B or C, and no readmission within 90 days, it was not common to achieve no postoperative major morbidities within 90 days. In our study, 42% of patients in the training cohort and 45% of patients in the external testing cohort failed to achieve TOLS, indicating that further research to improve the short-term outcomes of this surgery is required. To predict the probability of achieving TOLS in GBC patients treated with curative-intent resection, a nomogram was constructed using clinically relevant factors based on a multicenter database and tested externally in a single center database. The nomogram showed good fit and prediction performance in both the training and external testing cohorts.

In traditional surgical oncology, postoperative short-term outcomes have usually been reported as a single outcome to include one of the following: morbidity, mortality, margin status, and length of hospital stay^5,22,23^. Although, such single outcomes can allow surgeons to make clinical decisions to enhance certain specific single outcomes, it inevitably is unable to include all significant and important single outcomes. In addition, from the perspective of patients, surgical performance should be judged on whether the outcomes are ‘meeting their ideal or not meeting their ideal’ based on ‘all or nothing’. The Institute of Medicine in 2001 stated that the ideal medical process should be safe, effective, patient-centered, timely, efficient, and equitable^24^. In this context, TO has been proposed and applied as a comprehensive, patient-centered measure to assess short-term outcomes in ICC^25^ and HCC^26^ patients undergoing liver surgery. However, previous reported studies on TO were based on the definition of TO by a small group of surgeons or even by a single surgeon, leading to these definations not universally accepted.

Recently, an international group of experts proposed the difinitions of TOLS, which can be applied to patients undergoing hepatectomy as part of the surgical operation^12^. Such a definition of TOLS covers both the perspectives of patients and operating surgeons. From the perspective of patients, some important surgical outcomes such as resection margin status can be overlooked because of their lack of clinical background^27^. In fact, achieving R0 resection is a very important factor affecting the long-term outcomes of GBC patients after surgery^28,29^. From the perspective of surgeons, TOLS represents the success of surgery as TOLS covers both the safety and effectiveness of the operation. From the perspective of hospitals, TOLS can be used to compare the quality of surgery among different surgeons and different hospitals. Hepatobiliary surgeons and institutions that can achieve high rates of TOLS can share their surgical and management experiences in promoting the quality of surgery. When compared with the previous definitions used for TO, TOLS has the following advantages: First, in the previous definitions of TO, perioperative transfusion has been considered as an important adverse short-term outcome^25,26^. However, there is only one retrospective study which confirmed perioperative blood transfusion to be a negative factor affecting survival and recurrence in GBC patients after surgery^30^, with the resultant lack of concrete and credible evidence on the effect of perioperative blood transfusion on survival and recurrence in GBC patients after surgery. As perioperative blood transfusion is commonly used in complex hepatectomies, especially in hemodynamically unstable patients, it should be freely used if needed to these patients^31^. Second, length of hospital stay has been used as an important outcome in previous definitions of TO^32,33^. However, length of hospital stay relates not only on functional recovery, but it often depends on cultural differences and the organization of healthcare systems among different countries and regions.

In our study, several factors were found to be independently associated with TOLS, including age less than or equal to 70 years, absence of preoperative jaundice (TB≤3 mg/dl), T1 stage disease, N0 stage disease, wedge hepatectomy, and no neoadjuvant therapy. Some of these factors are particularly important to surgeons because they can be improved to increase the probability of achieving TOLS in the preoperative period.

Preoperative jaundice has also been shown to be associated with a higher incidence of postoperative morbidities by a previous study^34^. Surgeons are able to improve obstructive jaundice with preoperative biliary drainage. Although the timing of preoperative drainage is still controversial as prolonged biliary drainage can lead to the progression of tumors.

With recent advances in drug research, neoadjuvant therapy has been used to downstage a variety of malignant tumors. At present, no study has clearly demonstrated the benefit of neoadjuvant therapy for resectable GBC, although the NCCN guidelines still recommend a course of neoadjuvant therapy for GBC patients with preoperative jaundice^3^. Our study did not support the routine use of neoadjuvant therapy for patients with resectable GBC.

Curative resection for GBC patients requires a very comprehensive preoperative surgical evaluation^35,36^. The NCCN guidelines clearly state that cholecystectomy, hepatectomy, and lymphadenectomy are required for resectable GBC^3^. Empirical major hepatectomy (right hemi-hepatectomy) does not improve the long-term survival of GBC patients but it increases the risk of postoperative morbidities^37,38^. Therefore, extended hepatectomy beyond segment 4B+5 resection is not recommended except for patients with special indications to achieve R0 resection. Our study showed wedge hepatectomy to achieve better TOLS than segment 4B+5 resection. However, a recent study showed that for T2 GBC patients, segment 4B+5 resection reduced the risk of recurrence compared with wedge resection^39^. The combined 4B+5 segment resection and neoadjuvant therapy reduces the probability of achieving TOLS, but it potentially produce better long-term survival outcomes. Thus, to determine the type of hepatectomy and whether to give neoadjuvant therapy for GBC patients, surgeons need further studies by conducting a comprehensive preoperative assessment to include the extent of tumor invasion, physiological status, and liver function of patients to identify patients with a high-risk of recurrence by using the GBC survival prediction model. For these patients, it is worthwhile to sacrifice their probability of achieving TOLS in exchange for better long-term survival outcomes of these patients.

Recently, nomograms as predictive tools have been used, based on some specific variables to predict the risk of achieving a specific outcome for individuals with a certain disease^25,40^. In our study, an online nomogram was developed and validated in predicting the probability of achieving TOLS in GBC patients treated with curative-intent resection. This nomogram demonstrated good predictive performance in both the training and the external testing cohorts in identifying patients before surgery who are at an increased risk for an adverse postoperative outcome, thus helping surgeons in improving their surgical decision-making.

This study has limitations. First, this is a retrospective study with its inherent biases. The baseline differences between the training and the external testing cohorts were apparent. However, this heterogeneity may also be a strength as our results tend to be closer to a real-world study, which may be more generalizable. Second, the study lacked data from Western populations. The applicability of our nomogram to Western populations has not been confirmed. We have tried to use the SEER data, but the key perioperative short-term outcomes were not available in that database. Further studies on Western populations need to be carried out to validate our nomogram and results. Third, patients undergoing laparoscopic and robotic surgery were excluded from this study. There was a lack of good clinical data on laparoscopic and robotic surgery for GBC patients, although studies comparing open hepatectomy with laparoscopic hepatectomy for other liver tumors have been reported to show laparoscopic hepatectomy to be associated with fewer postoperative complications and shorter hospital stays^41,42^. Therefore, separate studies on patients receiving open or laparoscopic surgery on GBC need to be carried out. Forth, recently, there have been some new approaches to measuring morbidity, such as the complication severity score and the comprehensive complication index. These methods are able to consider all factors related to morbidity and generate a composite score, making outcomes easier to compare^43,44^. If the TOLS definition requires updating in the future, comprehensive complication index or complication severity score could be viable options for measuring morbidity. In addition, we should note that the Clavien–Dindo classification was considered valid only up to 30 days when it was initially used, but in 2018, it was validated from a clinical standpoint for up to 90 days^45^, and in 2019 from an economic standpoint^46^.

## Conclusions

More than half of the GBC patients treated with curative-intent resection in this study achieved TOLS. Our nomogram well predicted the probability of achieving TOLS before surgery, which can help surgeons to make better clinical decisions in managing and selecting their patients for surgery.

## Ethical approval

The study procedures were approved by the institutional ethics committee of Southwest Hospital (No. KY2022217).

## Funding

This work was supported in part by the National Natural Science Foundation of China (No. 81874211) and Chongqing Technology Innovation and Application Development Special Key Project (No. CSTC2021jscx-gksb-N0009).

## Author contribution

Z.-P.L.: conceptualization, formal analysis, methodology, writing-original draft; W.G.: data curation, formal analysis, methodology, writing-review and editing; D.-L.Y.: data curation, formal analysis, methodology, writing review and editing; W.-Y.C.: formal analysis, methodology, software and visualization; J.-Y.W.: software and visualization; X.-L.L.: data curation, methodology; P.Y.: data curation; C.Y.: data curation; Z.-P.W.: data curation; R.D.: data curation; Y.Z.: data curation; F.H.: data curation; J.-X.Z.: data curation; D.Z.: data curation; W.C.: data curation; Y.J.: data curation; J.B.: data curation; J.-J.W.: data curation; Y.-Q.Z.: formal analysis, methodology, supervision; H.-S.D.: conceptualization, supervision, writing-original draft; W.Y.L.: supervision, writing-review and editing; Z.-Y.C.: conceptualization, funding acquisition, writing-review and editing.

## Conflicts of interest

There are no conflicts of interest.

## Research registration unique identifying number (UIN)

1. Name of the registry: Research Registry
2. Unique Identifying number or registration ID: researchregistry8541
3. Hyperlink to your specific registration (must be publicly accessible and will be checked): https://www.researchregistry.com/browse-theregistry#home/registrationdetails/6394009a5269ee002646ddf2/

## Guarantor

Zhi-Yu Chen, Wan Yee Lau, Hai-Su Dai.

## Data availability statement

The data that support the study findings are available upon reasonable request from the corresponding authors (Zhi-Yu Chen, Wan Yee Lau, Hai-Su Dai). The full data are not publicly available due to limitations posed by the ethical regulations at some of the participating centers.

## Provenance and peer review

Not commissioned, externally peer-reviewed.

## Supplementary Material

SUPPLEMENTARY MATERIAL

## References

[1] Baiu I Visser B . Gallbladder cancer. JAMA 2018;320:1294. 30264121 10.1001/jama.2018.11815

[2] Rawla P Sunkara T Thandra KC . Epidemiology of gallbladder cancer. Clin Exp Hepatol 2019;5:93–102. 31501784 10.5114/ceh.2019.85166 PMC6728871

[3] Benson AB D’Angelica MI Abbott DE . Hepatobiliary cancers, version 2.2021, NCCN clinical practice guidelines in oncology. J Natl Compr Canc Netw 2021;19:541–565. 34030131 10.6004/jnccn.2021.0022

[4] McKay A Katz A Lipschitz J . A population-based analysis of the morbidity and mortality of gallbladder surgery in the elderly. Surg Endosc 2013;27:2398–2406. 23443477 10.1007/s00464-012-2746-x

[5] Dimick JB Welch HG Birkmeyer JD . Surgical mortality as an indicator of hospital quality: the problem with small sample size. JAMA 2004;292:847–851. 15315999 10.1001/jama.292.7.847

[6] Dimick JB Staiger DO Baser O . Composite measures for predicting surgical mortality in the hospital. Health Aff (Millwood) 2009;28:1189–1198. 19597221 10.1377/hlthaff.28.4.1189

[7] Rothberg MB Morsi E Benjamin EM . Choosing the best hospital: the limitations of public quality reporting. Health Aff (Millwood) 2008;27:1680–1687. 18997226 10.1377/hlthaff.27.6.1680

[8] Dimick JB Staiger DO Osborne NH . Composite measures for rating hospital quality with major surgery. Health Serv Res 2012;47:1861–1879. 22985030 10.1111/j.1475-6773.2012.01407.x PMC3448279

[9] Dimick JB Staiger DO Hall BL . Composite measures for profiling hospitals on surgical morbidity. Ann Surg 2013;257:67–72. 23235395 10.1097/SLA.0b013e31827b6be6

[10] Kolfschoten NE Kievit J Gooiker GA . Focusing on desired outcomes of care after colon cancer resections; hospital variations in ‘textbook outcome’. Eur J Surg Oncol 2013;39:156–163. 23102705 10.1016/j.ejso.2012.10.007

[11] Merath K Chen Q Bagante F . Textbook outcomes among medicare patients undergoing hepatopancreatic surgery. Ann Surg 2020;271:1116–1123. 30499800 10.1097/SLA.0000000000003105

[12] Görgec B Cacciaguerra AB Pawlik TM . An international expert delphi consensus on defining textbook outcome in liver surgery (TOLS). Ann Surg 2022;277:821–828. 35946822 10.1097/SLA.0000000000005668 PMC10082050

[13] Mathew G Agha R Albrecht J . STROCSS Group. STROCSS 2021: strengthening the reporting of cohort, cross-sectional and case-control studies in surgery. Int J Surg 2021;96:106165. 34774726 10.1016/j.ijsu.2021.106165

[14] Nagino M DeMatteo R Lang H . Proposal of a new comprehensive notation for hepatectomy: the “New World” terminology. Ann Surg 2021;274:1–3. 33630445 10.1097/SLA.0000000000004808

[15] Maker AV Butte JM Oxenberg J . Is port site resection necessary in the surgical management of gallbladder cancer. Ann Surg Oncol 2012;19:409–417. 21698501 10.1245/s10434-011-1850-9

[16] Giannis D Cerullo M Moris D . Validation of the 8th Edition American Joint Commission on Cancer (AJCC) gallbladder cancer staging system: prognostic discrimination and identification of key predictive factors. Cancers (Basel) 2021;13:547. 33535552 10.3390/cancers13030547 PMC7867111

[17] Yang XW Chen JY Wen ZJ . Effect of preoperative jaundice on long-term prognosis of gallbladder carcinoma with radical resection. World J Surg Oncol 2020;18:239. 32891147 10.1186/s12957-020-02015-2 PMC7487893

[18] Kazaryan AM Røsok BI Edwin B . Morbidity assessment in surgery: refinement proposal based on a concept of perioperative adverse events. ISRN surgery 2013;2013:625093. 23762627 10.1155/2013/625093 PMC3671541

[19] Koch M Garden OJ Padbury R . Bile leakage after hepatobiliary and pancreatic surgery: a definition and grading of severity by the international study group of liver surgery. Surgery (Oxf) 2011;149:680–688. 10.1016/j.surg.2010.12.002 21316725

[20] Rahbari NN Garden OJ Padbury R . Posthepatectomy liver failure: a definition and grading by the International Study Group of Liver Surgery (ISGLS). Surgery (Oxf) 2011;149:713–724. 10.1016/j.surg.2010.10.001 21236455

[21] Clavien PA Barkun J de Oliveira ML . The Clavien-Dindo classification of surgical complications: five-year experience. Ann Surg 2009;250:187–196. 19638912 10.1097/SLA.0b013e3181b13ca2

[22] Peterson ED Coombs LP DeLong ER . Procedural volume as a marker of quality for CABG surgery. JAMA 2004;291:195–201. 14722145 10.1001/jama.291.2.195

[23] Liu ZP Chen WY Zhang YQ . Postoperative morbidity adversely impacts oncological prognosis after curative resection for hilar cholangiocarcinoma. World J Gastroenterol 2022;28:948–960. 35317056 10.3748/wjg.v28.i9.948 PMC8908289

[24] Crossing the Quality Chasm: A New Health System for the 21st Century. Janet M. Corrigan, Molla S. Donaldson, and Lyla M. Hernandez. 2001 National Academy Press. 25057539

[25] Merath K Chen Q Bagante F . A multi-institutional international analysis of textbook outcomes among patients undergoing curative-intent resection of intrahepatic cholangiocarcinoma. JAMA Surg 2019;154:e190571. 31017645 10.1001/jamasurg.2019.0571 PMC6487899

[26] Liu ZP Yao LQ Diao YK . Association of preoperative body mass index with surgical textbook outcomes following hepatectomy for hepatocellular carcinoma: a multicenter study of 1206 patients. Ann Surg Oncol 2022;29:4278–4286. 10.1245/s10434-022-11721-y 35419755

[27] Marshall MN Shekelle PG Leatherman S . The public release of performance data: what do we expect to gain? A review of the evidence. JAMA 2000;283:1866–1874. 10770149 10.1001/jama.283.14.1866

[28] Littau MJ Kulshrestha S Bunn C . Is positive histologic surgical margin associated with overall survival in patients with resectable gallbladder cancer. Surg Open Sci 2021;6:15–20. 34409279 10.1016/j.sopen.2021.07.003 PMC8363875

[29] Vega EA Vinuela E Sanhueza M . Positive cystic duct margin at index cholecystectomy in incidental gallbladder cancer is an important negative prognosticator. Eur J Surg Oncol 2019;45:1061–1068. 30704808 10.1016/j.ejso.2019.01.013

[30] Lopez-Aguiar AG Ethun CG McInnis MR . Association of perioperative transfusion with survival and recurrence after resection of gallbladder cancer: A 10-institution study from the US Extrahepatic Biliary Malignancy Consortium. J Surg Oncol 2018;117:1638–1647. 29761515 10.1002/jso.25086 PMC10182890

[31] Li Z Sun YM Wu FX . Controlled low central venous pressure reduces blood loss and transfusion requirements in hepatectomy. World J Gastroenterol 2014;20:303–309. 24415886 10.3748/wjg.v20.i1.303 PMC3886023

[32] Wiseman JT Ethun CG Cloyd JM . Analysis of textbook outcomes among patients undergoing resection of retroperitoneal sarcoma: A multi-institutional analysis of the US Sarcoma Collaborative. J Surg Oncol 2020;122:1189–1198. 32696475 10.1002/jso.26136

[33] Sweigert PJ Eguia E Baker MS . Assessment of textbook oncologic outcomes following pancreaticoduodenectomy for pancreatic adenocarcinoma. J Surg Oncol 2020;121:936–944. 32124437 10.1002/jso.25861

[34] Yang XW Yuan JM Chen JY . The prognostic importance of jaundice in surgical resection with curative intent for gallbladder cancer. BMC Cancer 2014;14:652. 25187159 10.1186/1471-2407-14-652 PMC4164789

[35] Regimbeau JM Fuks D Bachellier P . Prognostic value of jaundice in patients with gallbladder cancer by the AFC-GBC-2009 study group. Eur J Surg Oncol 2011;37:505–512. 21514090 10.1016/j.ejso.2011.03.135

[36] Nishio H Ebata T Yokoyama Y . Gallbladder cancer involving the extrahepatic bile duct is worthy of resection. Ann Surg 2011;253:953–960. 21490453 10.1097/SLA.0b013e318216f5f3

[37] Foster JM Hoshi H Gibbs JF . Gallbladder cancer: defining the indications for primary radical resection and radical re-resection. Ann Surg Oncol 2007;14:833–840. 17103074 10.1245/s10434-006-9097-6

[38] Pawlik TM Gleisner AL Vigano L . Incidence of finding residual disease for incidental gallbladder carcinoma: implications for re-resection. J Gastrointest Surg 2007;11:1478–1486; discussion 1486-1487. 17846848 10.1007/s11605-007-0309-6

[39] Chen M Cao J Xiang Y . Hepatectomy strategy for T2 gallbladder cancer between segment IVb and V resection and wedge resection: a propensity score-matched study. Surgery (Oxf) 2021;169:1304–1311. 10.1016/j.surg.2020.12.039 33551070

[40] Liu ZP Chen WY Wang ZR . Development and validation of a prognostic model to predict recurrence-free survival after curative resection for perihilar cholangiocarcinoma: a multicenter study. Front Oncol 2022;12:849053. 35530316 10.3389/fonc.2022.849053 PMC9071302

[41] Dagher I Proske JM Carloni A . Laparoscopic liver resection: results for 70 patients. Surg Endosc 2007;21:619–624. 17285378 10.1007/s00464-006-9137-0

[42] Troisi R Montalti R Smeets P . The value of laparoscopic liver surgery for solid benign hepatic tumors. Surg Endosc 2008;22:38–44. 17705077 10.1007/s00464-007-9527-y

[43] De la Plaza Llamas R Ramia JM . Postoperative complications in gastrointestinal surgery: a “hidden” basic quality indicator. World J Gastroenterol 2019;25:2833–2838. 31249442 10.3748/wjg.v25.i23.2833 PMC6589738

[44] De la Plaza Llamas R Ramia JM . Cost of postoperative complications: how to avoid calculation errors. World J Gastroenterol 2020;26:2682–2690. 32550746 10.3748/wjg.v26.i21.2682 PMC7284181

[45] de la Plaza Llamas R Ramia Ángel JM Bellón JM . Clinical validation of the comprehensive complication index as a measure of postoperative morbidity at a surgical department: a prospective study. Ann Surg 2018;268:838–844. 30303875 10.1097/SLA.0000000000002839

[46] de la Plaza Llamas R Hidalgo VÁ Latorre Fragua RA . The cost of postoperative complications and economic validation of the comprehensive complication index: prospective study. Ann Surg 2021;273:112–120. 30985367 10.1097/SLA.0000000000003308

