# Supplementary material for: Textbook outcomes in liver surgery for gallbladder cancer patients treated with curative-intent resection: a multicenter observational study
Source: Int J Surg. 2023 Jun 5;109(9):2751–61. doi: 10.1097/JS9.0000000000000510 (PMC10498895; doi:10.1097/JS9.0000000000000510)
Supplement: SUPPLEMENTARY MATERIAL [file js9-109-2751-s001.docx]

**Supplemental** **Table 3.** The score value for each factor and calculation formula.

| Factors | Categories | Score value |
| --- | --- | --- |
| Age | ≤ 70 years | 46.87 |
|  | > 70 years | 0.00 |
| Preoperative jaundice (preoperative TB) | No (≤ 3 mg/dL) | 35.41 |
|  | Yes (> 3 mg/dL) | 0.00 |
| T stage | T1 | 100.00 |
|  | T2 | 66.18 |
|  | T3/T4 | 0.00 |
| N stage | N0 | 89.42 |
|  | N1 | 60.48 |
|  | N2 | 0.00 |
| Type of hepatectomy | Wedge hepatectomy | 46.60 |
|  | Segment IVB+V resection | 16.17 |
|  | Right hemi hepatectomy | 0.00 |
| Neoadjuvant therapy | No | 50.45 |
|  | Yes | 0.00 |

Total score = Age + Preoperative jaundice (preoperative TB) + T stage + N stage + Type of hepatectomy + Neoadjuvant therapy.

**Abbreviations:** TB, total bilirubin.
