# Supplementary figures and images for: Textbook outcomes in liver surgery for gallbladder cancer patients treated with curative-intent resection: a multicenter observational study
Source: Int J Surg. 2023 Jun 5;109(9):2751–61. doi: 10.1097/JS9.0000000000000510 (PMC10498895; doi:10.1097/JS9.0000000000000510)

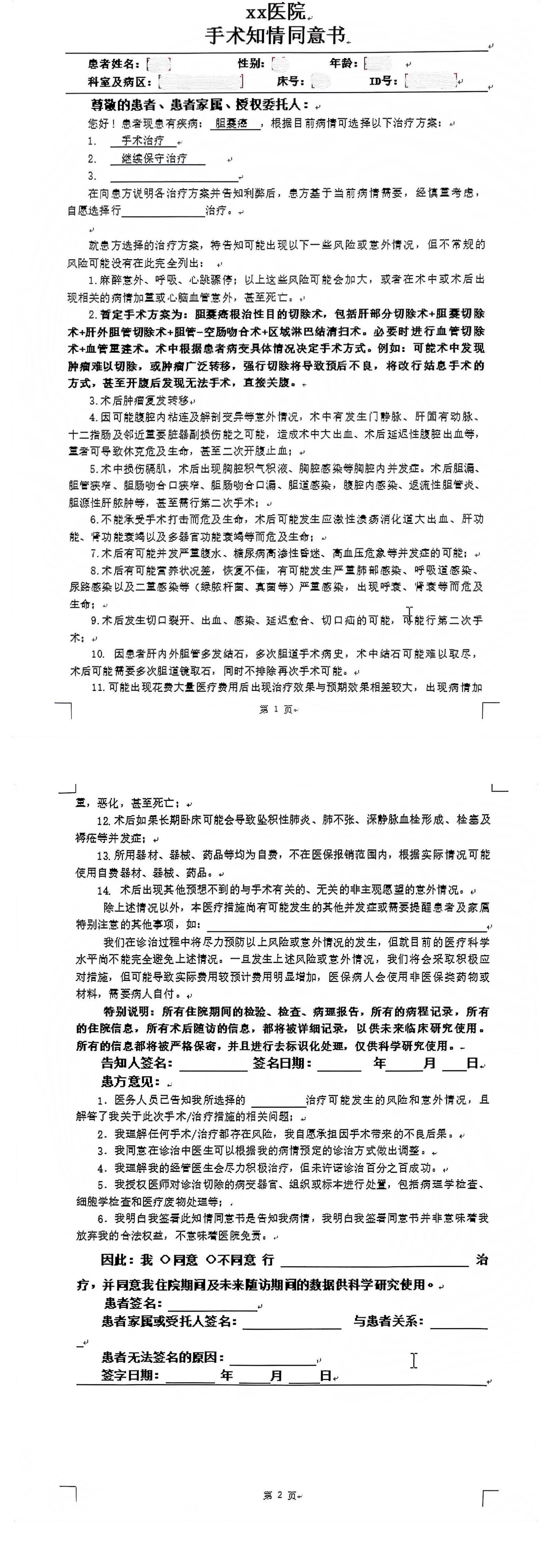

Supplement: SUPPLEMENTARY MATERIAL [file js9-109-2751-s003.jpg]
