# Supplementary material for: Textbook outcomes in liver surgery for gallbladder cancer patients treated with curative-intent resection: a multicenter observational study
Source: Int J Surg. 2023 Jun 5;109(9):2751–61. doi: 10.1097/JS9.0000000000000510 (PMC10498895; doi:10.1097/JS9.0000000000000510)
Supplement: SUPPLEMENTARY MATERIAL [file js9-109-2751-s004.docx]

**Supplemental Table 1.** Probability of each short-term outcome for GBC in the training and test cohorts.

| Short-term outcome | Training cohort (N=309) | Test cohort (N=128) |
| --- | --- | --- |
| Textbook outcome in liver surgery (TOLS) | 54.4% (168/309) | 57.8% (74/128) |
| No intraoperative grade ≥ 2 incidents | 95.5% (295/309) | 98.4% (126/128) |
| No postoperative bile leak of grade B or C | 86.4% (267/309) | 89.8% (115/128) |
| No postoperative liver failure grade B or C | 96.8% (299/309) | 96.1% (123/128) |
| No postoperative major morbidities within 90 days | 74.4% (230/309) | 72.7% (93/128) |
| No readmission within 90 days after discharge due to major morbidities | 94.5% (292/309) | 93.8% (120/128) |
| No mortality within 90 days | 84.1% (260/309) | 84.4% (108/128) |
| R0 resection | 91.9% (284/309) | 86.7% (110/128) |
| Length of hospital stay, day* | 14.5 ± 8.9 | 15.4 ± 9.1 |
| No perioperative blood transfusion | 75.1% (232/309) | 69.5% (89/128) |

**Note:** * Continuous values are expressed as the mean ± standard deviation.
