# Supplementary material for: Textbook outcomes in liver surgery for gallbladder cancer patients treated with curative-intent resection: a multicenter observational study
Source: Int J Surg. 2023 Jun 5;109(9):2751–61. doi: 10.1097/JS9.0000000000000510 (PMC10498895; doi:10.1097/JS9.0000000000000510)
Supplement: SUPPLEMENTARY MATERIAL [file js9-109-2751-s005.docx]

**Supplemental Table 2.** Baseline characteristics for GBC between TOLS and Non TOLS group in test cohort.

| Variables | Total (N=128) | TOLS (N=74) | Non TOLS (N=54) | *P* value |
| --- | --- | --- | --- | --- |
| Age, years* | 58.0 ± 10.4 | 58.6 ± 10.7 | 57.3 ± 10.2 | 0.486 |
| Male | 49 (38.3) | 29 (39.2) | 20 (37.0) | 0.805 |
| ASA score > II grade | 3 (2.3) | 1 (1.4) | 2 (3.7) | 0.385 |
| Comorbidity | 35 (27.3) | 25 (33.8) | 10 (10.85) | 0.056 |
| Preoperative jaundice | 48 (37.5) | 21 (28.4) | 27 (50.0) | 0.013 |
| Preoperative PTCD | 16 (12.5) | 10 (13.5) | 6 (11.1) | 0.685 |
| Incidentally discovered | 9 (7.0) | 6 (8.1) | 3 (5.6) | 0.577 |
| TB, umol/ml* | 16.5 (12.0, 114.4) | 15.6 (12.3, 86.4) | 45.3 (11.2, 207.4) | 0.086 |
| Albumin, g/L* | 39.2 ± 6.7 | 40.7 ± 7.0 | 37.1 ± 5.7 | 0.002 |
| ALT, U/L* | 41.5 (19.5, 102.2) | 36.3 (18.0, 89.2) | 61.5 (34.4, 113.8) | 0.303 |
| INR* | 0.98 ± 0.09 | 0.97 ± 0.08 | 0.99 ± 0.09 | 0.139 |
| CEA, ug/ml* | 2.5 (1.5, 4.7) | 2.4 (1.4, 4.5) | 2.9 (1.9, 6.1) | 0.108 |
| CA 19-9, U/L* | 33.5 (8.5, 249.5) | 25.2 (8.5, 201.4) | 74.0 (8.2, 279.0) | 0.368 |
| Tumor size, mm* | 23.5 (15.0, 40.0) | 20.0 (15.8, 41.2) | 25.0 (15.0, 40.0) | 0.734 |
| Poor differentiation | 39 (30.5) | 22 (29.7) | 17 (31.5) | 0.832 |
| Adenocarcinoma | 107 (83.6) | 65 (87.8) | 42 (77.8) |  |
| 8th AJCC T stage |  |  |  | 0.001 |
| T1 | 46 (35.9) | 34 (45.9) | 12 (22.2) |  |
| T2 | 62 (48.4) | 35 (47.3) | 27 (50.0) |  |
| T3/T4 | 20 (15.6) | 5 (6.8) | 15 (27.8) |  |
| 8th AJCC N stage |  |  |  | 0.030 |
| N0 | 69 (53.9) | 47 (63.5) | 22 (40.7) |  |
| N1 | 44 (34.4) | 19 (25.7) | 25 (46.3) |  |
| N2 | 15 (11.7) | 8 (10.8) | 7 (13.0) |  |
| 8th AJCC staging system |  |  |  | 0.035 |
| I stage | 33 (25.8) | 26 (35.1) | 7 (13.0) |  |
| II stage | 29 (22.7) | 16 (21.6) | 13 (24.1) |  |
| III stage | 49 (38.3) | 23 (31.1) | 26 (48.1) |  |
| IV stage | 17 (13.3) | 9 (12.2) | 8 (14.8) |  |
| Type of hepatectomy |  |  |  | 0.796 |
| Wedge hepatectomy | 87 (68.0) | 52 (70.3) | 35 (64.8) |  |
| Segment IVB + V resection | 20 (15.6) | 11 (14.9) | 9 (16.7) |  |
| Right hemi hepatectomy | 21 (16.4) | 11 (14.9) | 10 (18.5) |  |
| Intraoperative blood loss, ml* | 300 (200, 500) | 300 (250, 500) | 350 (200, 600) | 0.561 |
| Bile duct procedure* | 89 (69.5) | 54 (73.0) | 35 (64.8) | 0.322 |
| Adjuvant therapy | 23 (18.0) | 13 (17.6) | 10 (18.5) | 0.890 |
| Neoadjuvant therapy | 13 (10.2) | 2 (2.7) | 11 (20.4) | 0.001 |

**Note:** * Continuous values are expressed as the mean ± standard deviation or median (quartile). ** The bile duct procedure included common bile duct resection and cholangiojejunostomy.

**Abbreviations:** AJCC, American Joint Committee on Cancer; ALT, alanine aminotransferase; ASA, American Society of Anesthesiologists; CA 19-9, carbohydrate antigen 19-9; CEA, carcinoembryonic antigen; INR, international normalized ratio; TB, total bilirubin.
