# Supplementary material for: Textbook outcomes in liver surgery for gallbladder cancer patients treated with curative-intent resection: a multicenter observational study
Source: Int J Surg. 2023 Jun 5;109(9):2751–61. doi: 10.1097/JS9.0000000000000510 (PMC10498895; doi:10.1097/JS9.0000000000000510)
Supplement: SUPPLEMENTARY MATERIAL [file js9-109-2751-s006.docx]

**Supplemental Table 4.** Comparison of the predictive ability for TOLS of the models.

| Accuracy index | Nomogram | 8th AJCC stage | *P* value |
| --- | --- | --- | --- |
|  | Training cohort | |  |
| AUC (95% CI) | 0.741 (0.689-0.789) | 0.628 (0.571-0.682) | < 0.001 |
| Sensitivity | 79.17% | 60.12% | -- |
| Specificity | 58.16% | 58.87% | -- |
| AIC | 379.50 | 412.78 | -- |
|  | Test cohort | |  |
| AUC (95% CI) | 0.726 (0.640-0.801) | 0.621 (0.531-0.705) | < 0.001 |
| Sensitivity | 70.27% | 35.14% | -- |
| Specificity | 68.52% | 87.04% | -- |
| AIC | 155.53 | 172.13 | -- |

**Abbreviations:** AIC, Akaike information criterion; AJCC, American Joint Committee on Cancer; AUC, area under the curve; CI, confidence interval.
